# Relationship Between Body-Specific Attention to a Paretic Limb and Real-World Arm Use in Stroke Patients: A Longitudinal Study

**DOI:** 10.3389/fnsys.2021.806257

**Published:** 2022-02-22

**Authors:** Ryoji Otaki, Yutaka Oouchida, Naoki Aizu, Tamami Sudo, Hiroshi Sasahara, Yuki Saito, Sunao Takemura, Shin-Ichi Izumi

**Affiliations:** ^1^Department of Physical Medicine and Rehabilitation, Tohoku University Graduate School of Medicine, Sendai, Japan; ^2^Department of Rehabilitation, Yamagata Saisei Hospital, Yamagata, Japan; ^3^Department of Education, Osaka Kyoiku University, Osaka, Japan; ^4^Faculty of Rehabilitation, School of Health Sciences, Fujita Health University, Toyoake, Japan; ^5^Department of Computer and Information Sciences, Tokyo University of Agriculture and Technology, Tokyo, Japan; ^6^Department of Neurosurgery, Yamagata Saisei Hospital, Yamagata, Japan; ^7^Department of Physical Medicine and Rehabilitation, Tohoku University Graduate School of Biomedical Engineering, Sendai, Japan

**Keywords:** accelerometer, body representation, body-specific attention, learned nonuse, recovery, rehabilitation, stroke, upper limb

## Abstract

Learned nonuse is a major problem in upper limb (UL) rehabilitation after stroke. Among the various factors that contribute to learned nonuse, recent studies have focused on body representation of the paretic limb in the brain. We previously developed a method to measure body-specific attention, as a marker of body representation of the paretic limb and revealed a decline in body-specific attention to the paretic limb in chronic stroke patients by a cross-sectional study. However, longitudinal changes in body-specific attention and paretic arm use in daily life (real-world arm use) from the onset to the chronic phase, and their relationship, remain unknown. Here, in a longitudinal, prospective, observational study, we sought to elucidate the longitudinal changes in body-specific attention to the paretic limb and real-world arm use, and their relationship, by using accelerometers and psychophysical methods, respectively, in 25 patients with subacute stroke. Measurements were taken at baseline (T_BL_), 2 weeks (T_2w_), 1 month (T_1M_), 2 months (T_2M_), and 6 months (T_6M_) after enrollment. UL function was measured using the Fugl-Meyer Assessment (FMA) and Action Research Arm Test (ARAT). Real-world arm use was measured using accelerometers on both wrists. Body-specific attention was measured using a visual detection task. The UL function and real-world arm use improved up to T_6M_. Longitudinal changes in body-specific attention were most remarkable at T_1M_. Changes in body-specific attention up to T_1M_ correlated positively with changes in real-world arm use up to T_6M_, and from T_1M_ to T_6M_, and the latter more strongly correlated with changes in real-world arm use. Changes in real-world arm use up to T_2M_ correlated positively with changes in FMA up to T_2M_ and T_6M_. No correlation was found between body-specific attention and FMA scores. Thus, these results suggest that improved body-specific attention to the paretic limb during the early phase contributes to increasing long-term real-world arm use and that increased real-world use is associated with the recovery of UL function. Our results may contribute to the development of rehabilitation strategies to enhance adaptive changes in body representation in the brain and increase real-world arm use after stroke.

## Introduction

The most common disability after stroke is upper limb (UL) paralysis, which occurs contralateral to a unilateral hemispheric injury. More than 80% of stroke patients experience this condition in the acute phase, and more than 40% of these patients have a residual disability in the chronic phase (Gresham et al., 1995). With the recent developments in acute stroke treatment, it has been reported that the percentage of patients with UL motor impairment within 72 h after stroke onset has decreased; however, 48% of stroke patients still had UL motor impairment (Alt Murphy et al., 2011; Persson et al., 2012). UL paralysis affects activities of daily living and reduces the quality of life (Nichols-Larsen et al., 2005). Many stroke patients with UL paralysis will stop using their paretic hand to avoid failure or inconvenience. Once the patient becomes accustomed to using the non-paretic hand, attempts to use the paretic hand are further reduced, resulting in learned nonuse, which is a major clinical problem (Taub et al., 2002). Learned nonuse leads to progressively smaller cortical areas representing the paretic limbs of the brain due to use-dependent neuroplasticity. These secondary changes in the neural system can worsen the motor impairment of the paretic limb. This negative cycle caused by learned nonuse prevents the recovery of the paretic limb after a stroke. For these reasons, in recent rehabilitation medicine, active use of the paretic limb is recommended to overcome and prevent learned nonuse (Morris et al., 2006).

Since the concept of learned nonuse was proposed, the importance of using the paretic hand in daily life (real-world arm use) as well as in function has been emphasized in the rehabilitation of stroke patients (Hebert et al., 2016; Winstein et al., 2016; Kelly et al., 2018). Accelerometers have been widely used as a method to measure real-world arm use objectively (Uswatte et al., 2000, 2005a). Early studies using accelerometers focused on stroke patients in the chronic phase (Uswatte et al., 2000, 2005a), but later cross-sectional studies investigated the acute phase (Gebruers et al., 2008) and the subacute phase (Thrane et al., 2011; Alt Murphy et al., 2019) of stroke. These studies using accelerometers from the acute to the chronic phase after stroke have been conducted and established an objective method to measure real-world arm use after stroke (Noorkoiv et al., 2014). Using accelerometers, several studies have reported factors associated with paretic hand use in patients with stroke. Although it has been thought that improvements in UL function translate directly to increased arm use in daily life, evidence from recent research, in which daily life arm use was evaluated using accelerometry, did not support this notion (Rand and Eng, 2012, 2015; Waddell et al., 2017). Instead, these studies indicated that, while UL function and arm use in daily life are related, they are distinctly different constructs. Specifically, it has been recognized that there is a disparity between UL function and real-world arm use (Rand and Eng, 2012). Despite improvements in UL function with inpatient rehabilitation after stroke, the use of the paretic hand did not improve significantly, and the use ratio of the paretic hand to the non-paretic hand was only 25%. Furthermore, when the effect of whether the paretic hand was dominant in real-world arm use was examined, no significant effect was found (Rand and Eng, 2012). In addition, this discrepancy between improved UL function and increased real-world arm use is known to occur even after hospital discharge (Rand and Eng, 2015; Doman et al., 2016). A comparison of paretic hand use at discharge to home and 12 months post-stroke showed that, despite significant improvement in UL function, there was no significant improvement in the use of the paretic hand in daily life. The use ratio of the paretic hand to the non-paretic hand at 12 months after stroke was 35%, which was very limited (Rand and Eng, 2015). These findings suggest that there is a discrepancy between improvement in UL function and real-world arm use and that factors other than function may also influence real-world arm use. Other studies have examined the impact of individual factors, including psychosocial factors and function, on real-world arm use. Observations up to 12 weeks after onset showed that real-world arm use increased, but that psychosocial factors (belief and confidence in UL performance, and motivation for UL use) had no effect on real-world arm use (Waddell et al., 2019a). A study of the psychosocial factors up to 6 months post-onset found that beliefs, confidence, and motivation regarding UL use remained high up to 6 months post-stroke, there was no correlation between psychosocial factors and clinical outcomes (Waddell et al., 2019b). Although these studies on real-world arm use have examined functional and psychosocial factors, the factors that influence the recovery of real-world arm use remain unclear.

In recent years, among the various factors that contribute to learned nonuse, many studies have focused on body consciousness, including body representation in the brain (Aymerich-Franch and Ganesh, 2016; Naito et al., 2016; Oouchida et al., 2016; Matamala-Gomez et al., 2020). Body consciousness is the consciousness of one’s own body, which has been discussed in philosophy and phenomenology. When body consciousness changes after a stroke, stroke patients relate the consciousness that “I feel as if it is not my hand” about the paretic limb. This body consciousness is broadly classified by Gallagher into “sense of body ownership” and “sense of agency” (Gallagher, 2000). In the hierarchy of body consciousness, sensorimotor representation is the lowest level. Sensorimotor representation is the body consciousness that is integrated by sensory and predictive information. This is the body representation in the brain that has been studied in the fields of psychology and medicine in recent years. Human movements are planned and executed based on this body representation in the brain.

In stroke patients, in addition to the paralysis caused by the brain injury, maladaptive changes in body representation of the paretic limb in the brain may prevent the use of the paretic limb in daily life. If the brain does not recognize the paretic hand as “my hand,” it may be difficult to use the paretic hand spontaneously in daily life. Body consciousness, including body representation of the limb in the brain, could not be observed externally. Thus, a method, using a visual detection task, has been developed to measure the amount of body-specific attention, i.e., attention specifically directed at the body (Aizu et al., 2018). In the above method, based on the body facilitation effect of visual detection of the self (Hari and Jousmäki, 1996; Whiteley et al., 2004, 2008; Reed et al., 2006, 2010; Tseng et al., 2012), the difference between the reaction time to a visual target outside the body and the reaction time to visual target on the body is defined as body-specific attention. This facilitation effect has been reported to occur even when the hand is not visible (Reed et al., 2006), and by attributing the hand to oneself (Whiteley et al., 2008). These studies support the idea that body representations in the brain facilitate the detection of visual information. In chronic stroke patients, it has been reported that, the more severe the hemiparesis and the longer the duration of the stroke, the lower is the body-specific attention (the facilitation effect did not occur in the paretic hand; Aizu et al., 2018). The results suggested that maladaptive changes in the body representation of the paretic hand in the brain occurred due to learned nonuse in chronic stroke patients. However, although the relationship between body-specific attention and UL function in cross-sectional studies of chronic stroke patients has been clarified, the longitudinal changes in body-specific attention and real-world arm use from stroke onset to the chronic phase, and the relationship between them, are unclear.

We hypothesized that improvements in body-specific attention to the paretic hand may facilitate real-world arm use. Since the previous study made it possible to measure learned nonuse from the aspect of body representation quantitatively, it may be possible to clarify how learned nonuse of the paretic limb progresses after the onset of stroke from the perspective of body representation in the brain and behavior of stroke patients, by using a visual detection task and accelerometer.

Therefore, this study aimed to elucidate the longitudinal changes in body-specific attention to the paretic limb and real-world arm use, and the relationship between them, by using accelerometers and psychophysical methods, respectively, in subacute stroke patients. This is expected to clarify the adaptive mechanisms underlying UL recovery after stroke from the perspective of behavior and body representation of the paretic limb in the brain.

## Materials and Methods

### Study Design

This was a longitudinal, prospective, observational cohort study involving patients with hemiparesis after stroke.

### Participants

This study collected data from the Yamagata Saisei Hospital, Japan. The inclusion criteria were: (1) first-ever stroke; (2) unilateral supratentorial lesions; (3) presence of UL motor deficits; (4) general condition was stable after 2 weeks from stroke onset; (5) age between 20 and 80 years. Patients with (1) multiple strokes; (2) aphasia; (3) apraxia; (4) attentional bias to the left or right visual field, which can be found in unilateral spatial neglect (USN); (5) hemianopia; or (6) serious uncontrolled medical conditions were excluded. From 2018 to 2020, 25 patients were recruited consecutively from the inpatients of Yamagata Saisei Hospital.

Thus, the subjects were 25 patients with subacute stroke whose general condition was stable after 2 weeks from stroke onset [mean age ± standard deviations (SD), 60.8 ± 12.8 years; 18 male; 13 right hemiparesis; 25 right-handed (Table 1)]. The measurement time points were baseline (within 1 week of study enrollment, T_BL_), 2 weeks (T_2w_: T_BL_+ 2 weeks), 1 month (T_1M_: T_BL_+ 1 month), 2 months (T_2M_: T_BL_+ 2 months) and 6 months (T_6M_: T_BL_+ 6 months) after the baseline measurement. For rehabilitation during the study period, the usual occupational and physical therapies were performed according to the general condition (early mobilization, activities of daily living training, and self-management guidance). The content and amount of UL exercises were controlled using a standardized UL program graded repetitive arm supplementary program (GRASP) for 1 h per day (Harris et al., 2009, 2010; Murdolo et al., 2017; Simpson et al., 2017).

**Table 1:** Clinical characteristics of stroke patients.

ID	Age(years)	Sex	Diagnosis	Lesion location	Duration (days)	Paresis side	Handedness	FMA-UE motor	FMA-UEID sensory	
S1	71	M	I	L Thalamus	53	R	R	14	6
S2	69	M	H	R Thalamus	40	L	R	41	6
S3	80	M	H	R Putamen	25	L	R	45	6
S4	59	F	H	L Thalamus	32	R	R	47	0
S5	61	M	I	L ICA	35	R	R	42	12
S6	35	M	I	R Putamen, Corona radiata	30	L	R	22	12
S7	66	M	I	L Corona radiata	36	R	R	38	10
S8	76	M	I	L Corona radiata	31	R	R	34	12
S9	71	M	H	R Putamen	19	L	R	53	10
S10	22	F	I	R ICA	60	L	R	15	8
S11	53	F	H	R Putamen	89	L	R	14	2
S12	49	M	I	R MCA	45	L	R	15	6
S13	58	M	I	R Putamen, Corona radiata	20	L	R	39	10
S14	73	F	I	R Corona radiata	31	L	R	19	12
S15	71	F	H	L Thalamus	39	R	R	20	0
S16	61	M	I	L Putamen, Corona radiata	42	R	R	33	10
S17	58	M	H	R Thalamus	56	L	R	28	7
S18	53	M	I	L Posterior limb of internal capsule	25	R	R	38	12
S19	66	M	I	R MCA	15	L	R	48	10
S20	72	F	I	L Posterior limb of internal capsule	20	R	R	53	10
S21	66	M	I	R Internal capsule, Corona radiata	18	L	R	21	10
S22	47	M	H	L Putamen	29	R	R	32	9
S23	58	M	H	L Putamen	25	R	R	14	10
S24	63	M	I	R Putamen, Corona radiata	39	L	R	37	12
S25	62	F	H	L Putamen	63	R	R	21	10
Mean	60.8	F: 7	I: 15	L: 12	36.7	R: 13	R: 25	31.3	8.5
SD	12.8	M: 18	H: 10	R: 13	17.1	L: 12	L: 0	12.8	3.5

*H, hemorrhage; I, infarction; ICA, internal carotid artery; MCA, middle cerebral artery; FMA, Fugl–Meyer assessment*.

### Standard Protocol Approvals, Registrations, and Patient Consents

The Tohoku University and Yamagata Saisei Hospital ethics committee approved this study (ID 2017-1-1076, ID348), which was conducted according to the ethical standards of the Declaration of Helsinki. Prior to our experiment, all participants agreed to participate in our experiment and provided written informed consent.

### Real-World Arm Use Measurement

Real-world arm use was measured using wrist-worn accelerometers (ActiGraph Link, GT9X, Actigraph Corp, Pensacola, FL). These wireless devices are small (35 mm × 35 mm × 10 mm, 14 g), have a solid-state accelerometer with a dynamic range of ±8 G gravity units, and can collect data locally. Wrist-worn accelerometers have an established validity and reliability for measuring the activity of the paretic limb in stroke patients (Uswatte et al., 2005b, 2006). Many methods of measuring paretic limb activity (equipment, measurement time) in stroke patients have been reported (Uswatte et al., 2005a; Noorkoiv et al., 2014; Hayward et al., 2016), and the measurement and data analysis methods used in this study were performed as previously reported (Uswatte et al., 2006; Taub et al., 2013).

Accelerometers were attached to both wrists. The accelerometer was attached to the distal forearm using a wristwatch-type belt (Actigraph, Pensacola, FL). In order to obtain accurate data, the accelerometer was initially attached by an occupational therapist who confirmed the appropriate belt length and position according to the circumference of the patient’s distal forearm (to avoid loosening). Over the 3-day measurement period (Van Der Pas et al., 2011; Franck et al., 2019), patients wore the accelerometers from waking until sleep, and were only removed from both wrists during bathing. Patients and caregivers were instructed to comply with the wearing method and wearing time described above, and all precautions were documented and given to them. In addition, to prevent inappropriate wearing and loss of data, ward nurses attached and removed the accelerometers during hospitalization, and family members assisted the patient in wearing and removing the accelerometers after discharge, except for patients who lived alone (caregivers always checked the accelerometers even for patients who could remove them by themselves). As a safety consideration, the presence of skin problems in the belt area was checked daily, and the presence of pain or discomfort was assessed daily. Data analysis was performed excluding the time when the accelerometer was not attached, such as sleeping and bathing, from the 72-h measurement period. Data were sampled and recorded along three axes (X, Y, and Z axes) at 60 Hz. The obtained data were band-pass filtered between 0.25 Hz and 2.5 Hz, and activity counts (acceleration unit set by Actigraph Corp) for each axis were collected in 60-s epochs, using ActiLife 6 software (Actigraph Corp). Activity counts were combined across the three axes to create a single-vector magnitude value (√x^2^ + y^2^ + z^2^) for each second of the data. The amount of use of the paretic hand in daily life was defined as real-world arm use (ratio of paretic to non-paretic vector magnitude value). A ratio close to 1.0 means that the paretic hand is used to the same extent as the non-paretic hand, and a ratio close to 0 indicates that the paretic hand is used markedly less frequently than the non-paretic hand (Uswatte et al., 2006; Taub et al., 2013; Gebruers et al., 2014; Hayward et al., 2016).

### Body-Specific Attention to the Paretic Hand

As an index reflecting the body representation of the paretic hand in the brain, we measured the body-specific attention of the paretic hand in a visual detection task (Aizu et al., 2018). Since the brain monitors the location of its own body parts by body representations in the brain, it is assumed that attention is more strongly directed to body space than to out-of-body space. Previous studies have reported a body-related “facilitation effect” that allows healthy subjects to detect a visual target faster when the target is presented near the hand than when it is presented far away (Hari and Jousmäki, 1996; Whiteley et al., 2004, 2008; Reed et al., 2006, 2010; Tseng et al., 2012). Such a facilitation effect has been used in psychophysical experiments as a reflection of body representations in the brain (Whiteley et al., 2008). For example, it has been shown that there is no facilitation effect in conditions where no attribution is given to the hand, but there is a facilitation effect in conditions where attribution is given to the hand by passive movement of the hand (Whiteley et al., 2008). This indicates that the facilitation effect occurs only when the brain recognizes the hand as part of its own body, suggesting that body representation in the brain facilitates the detection of visual information. It was also shown that the facilitation effect occurred even in the condition of only proprioceptive information where the hand could not be visually confirmed (Reed et al., 2006). Furthermore, in a study on the facilitation effects of tools, it was reported that training with a rake in hand facilitated the detection of targets presented on the rake (Kao and Goodale, 2009). This result suggests that the tool is incorporated as the body representation in the brain, i.e., the brain has come to recognize the tool as a part of its own body. This result strongly supports the idea that the body facilitation effect reflects the body representation in the brain. In addition, a recent study by Aizu reported that healthy subjects responded more quickly to visual targets on their own hand using a visual detection task designed to measure body facilitation effects (Aizu et al., 2018). This facilitative effect of visual detection on one’s own body has been interpreted as a result of latent attention to one’s own body and is referred to as body-specific attention (Aizu et al., 2018). Body-specific attention, which is the attention potentially directed to one’s own body, may reflect body representations in the brain.

In the present study, we measured the body-specific attention to the paretic hand in a visual detection task as an index reflecting the body representation of the paretic limb in the brain (Aizu et al., 2018).

#### Experimental Setup and the Visual Detection Task

**Figure 1:**
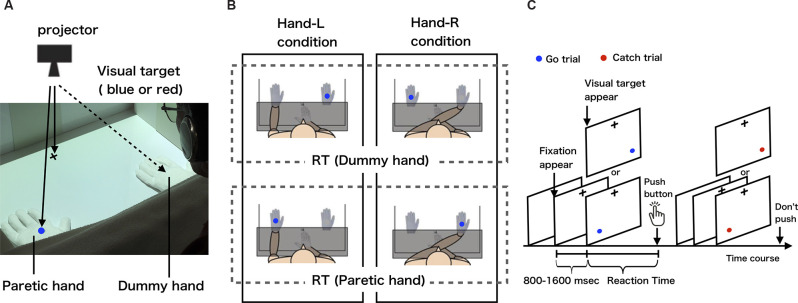
**(A)** The measurement of body-specific attention to the paretic hand. **(B)** Experimental conditions and definition of body-specific attention (each condition is circled by a solid line; dummy or paretic hand reaction time is circled by a dotted-line). These schematic views show the hand-L and hand-R conditions for patients with left hemiparesis. The patients placed their paretic hand either on the left side (hand-L) or right side (hand-R). In both conditions, the dummy hand was placed on the opposite side of the midline to the paretic hand. For patients with right hemiparesis, the paretic hand was placed to the left of the midline in the hand-L condition and to the right of the midline in the hand-R condition. We defined the amount of body-specific attention directed to the paretic hand as the average reaction time for when the visual target was presented on the dummy hand minus the average reaction time for when the visual target was presented on the paretic hand (body-specific attention = dummy hand − paretic hand). **(C)** Visual detection task. After the presentation of a fixation point, the visual target appeared randomly from 800 to 1,600 ms, on one of the two hands (paretic or dummy hand). Participants pushed the button only when a blue-filled circle appeared.

The visual detection task was created in the programming software MATLAB (version 2017b, MathWorks, Natick, MA, USA) using a Macintosh personal computer (Mac Book Pro, Apple Inc., Cupertino, CA, USA). In the visual detection task, a fixation point was first presented in the center of the screen, and then the button (USB numeric keypad, SANWA SUPPLY NT-9UH2BK, Okayama, Japan) was pressed as soon as possible when the blue visual target (Go trial) appeared in either the left or right position (Figures 1A–C). In this case, the button was not pressed when the red visual target (Catch trial) appeared (Figure 1C). The time between the presentation of the visual target and pressing of the button was recorded as the reaction time. To exclude the effect of anticipation of the appearance of the visual target on the reaction time, the time from the fixation point to the presentation of the visual target was varied randomly between 800 and 1,600 ms, and the visual target was presented randomly in either the left or right position. In addition, blue or red visual targets were randomly presented at a ratio of 4:1.

The subjects sat in a chair in front of a desk in a quiet room. A personal computer on which the visual detection task was created was connected to a projector (EB-S12H, Epson, Suwa, Japan) set up above the desk such that a fixation point and visual target could be projected onto the desk (Figure 1A). Responsive buttons were placed at the center of the desk (Figure 1B). The visual target had a visual angle of 1.7°, 28 cm away from the subject, with 36 cm between the visual targets. The distance between the projected visual targets and the fixation point was 28 cm. To ensure that the reaction time results were not affected by eye movements, subjects were instructed to gaze at the fixation point throughout the task. Considering the effects of fatigue during the experiment, the number of visual targets presented in a single condition was set to 80. In order to control the visual information given to the subject during the experiment, the two hands in which the visual target was presented were a paretic hand wearing white cotton gloves and a dummy hand made of white cotton gloves (Figures 1A,B). In addition, a whiteboard was used to hide the subject’s forearm and button-pressing hand from the subject’s view during the task, thus controlling the visual information given to the subject.

#### Experimental Condition

To measure the body-specific attention directed at the paretic hand quantitatively, we set up two experimental conditions and one control condition. In the experimental condition, in order to exclude the effect of hand position in space, we set the hand-L condition, in which the paretic hand was placed in the left position and the dummy hand in the right position (Figure 1B left), and the hand-R condition, in which the paretic hand was placed in the right position and the dummy hand in the left position (Figure 1B right), at the two locations at which the visual target was presented on the desk. In the control condition, the dummy hand was placed at both locations where the visual target was presented on the desk (the paretic hand was on the abdomen), in order to check for attentional bias in space. Button pressing was performed with the index finger on the non-paretic side. For example, when a left hemiplegic patient performed the experimental condition (hand-L condition), the paretic hand was wearing a white cotton glove, the paretic hand was placed in the space on the left side, and the button was pressed with the right, non-paretic hand (Figures 1A,B). The experiment was counterbalanced for each subject so that the order of task execution did not affect the results. To familiarize the subjects with the visual detection task, we presented 60 visual targets and had participants perform the task as practice trials. In the practice trials, the subject’s paretic hand or dummy hand was not placed on the desk, but the visual target was presented directly on the desk and the subject responded. The subjects were asked to perform the task without any instructions that could direct their attention intentionally.

#### Experimental Data Analysis

The average reaction time of paretic and dummy hands was calculated for each condition to ascertain the amount of body-specific attention to the paretic hand (Figure 1B). To remove the effects of delayed reactions due to inattention during the task and accelerated reactions due to anticipation of the visual target appearing, the obtained reaction time data exceeding two SD above and below the mean reaction time were excluded from the results as outliers.

Based on the results obtained under the experimental conditions, we defined the amount of body-specific attention directed to the paretic hand as the average reaction time for when the visual target on the dummy hand was presented minus the average reaction time for when the visual target on the paretic hand was presented (body-specific attention = dummy hand − paretic hand). A positive value of body-specific attention means that the reaction time to the presentation of the visual target on the paretic hand was shorter than the reaction time to the presentation of the visual target on the dummy hand.

### Additional Study Assessments

The Fugl-Meyer Assessment (FMA) was used to evaluate clinical UL function. The FMA is a stroke-specific rating scale based on the recovery process of hemiplegia after stroke. The FMA upper extremity motor (FMA-UE motor) consists of 33 items, each of which is scored on a scale of 0–2 (0 = generally corresponding to no function, 1 = partial function, 2 = perfect function), with the highest score being 66 (Fugl-Meyer et al., 1975). The Action Research Arm Test (ARAT) was used to measure UL capacity. The ARAT is a valid and reliable measure of UL capacity in adults with paresis. It is a 19-item assessment of grasp, grip, pinch, and gross motor function. Individual items were scored using a 0–3 ordinal scale (0 = can perform no part of the test, 1 = performs test partially, 2 = completes test but takes abnormally long time or has great difficulty, 3 = performs test normally). Individual item scores are summed, and the final score ranges from 0 to 57, with higher scores indicating better motor function (Lyle, 1981; Van der Lee et al., 2001).

The FMA-UE sensory score was measured as a sensory function assessment. FMA-UE sensory consists of six items: light touch (forearm, palm) and proprioception (shoulder, elbow, wrist, thumb IP), and each item is scored on a 3-point scale from 0 to 2 (light touch score 0 = anesthesia; 1 = hypoesthesia or dysesthesia; 2 = normal, proprioception score 0 = less than 3/4 of the answers correct or absence; 1 = 3/4 of the answers correct or considerable difference; 2 = correct 100% or no difference). The maximum score is 12 points (Fugl-Meyer et al., 1975).

### Statistical Analysis

To examine longitudinal changes in UL function, real-world arm use and body-specific attention were analyzed using repeated measures analysis of variance (ANOVA) and multiple comparisons were adjusted with the Bonferroni correction. To examine longitudinal changes in sensory function (FMA-UE sensory) was analyzed using Friedman test and multiple comparisons were adjusted with the Bonferroni correction. For comparison between two groups of stroke patients in this study and healthy older people in a previous study that used cross-sectional data, at each time point of body-specific attention, we used the Welch’s two-sample *t*-test (data of healthy older people were taken from a previous study by Aizu et al. (2018).

To investigate the correlation between the amount of change in real-world arm use and the amount of change in body-specific attention during the UL recovery process, Pearson’s product-moment correlation coefficient or Spearman’s rank correlation coefficient tests were performed, after assessing the normality of data distribution by using the Shapiro–Wilk test. This correlation analysis also focused on the amount of change in each index over different time periods in order to clarify the hypothesis that increased body-specific attention would promote real-world arm use. Specifically, we examined the relationship between the amount of change in body-specific attention in the early phase and the amount of change in real-world arm use until the chronic phase, because plastic changes in the brain, including body representations, change significantly in the early phase after stroke onset, but behavioral changes, such as arm use, may require a longer time. To examine the patient-specific factors affecting changes in body-specific attention, as well as changes in real-world arm use, characteristics at enrollment, such as age, duration since onset, FMA-UE sensory, and FMA-UE motor were investigated using Pearson’s product-moment correlation coefficient or Spearman’s rank correlation coefficient test, after assessing data normality by the Shapiro–Wilk test. Based on the principle of use-dependent plasticity of the brain, we also examined the relationship between the amount of change in real-world arm use and the amount of change in UL function. It was considered that if real-world arm use improved in the early phase, UL function would recover in the long term, but conversely, if real-world arm use decreased in the early phase, functional recovery in the long term would be poor. Therefore, we focused on such periods to conduct a correlation analysis between arm use and UL function. Although our main interest was in the analysis of longitudinal data, correlations in cross-sectional data were also analyzed using the same procedure, in order to examine the relationship between indices at each time point. The statistical significance level was set at 5%. Statistical analyses were performed using SPSS, version 27.0 J (SPSS Japan Inc., Tokyo, Japan).

## Results

There were no participant dropouts during the 6-month study period. All outcomes were measured in five assessment sessions for all patients.

### UL Function (FMA-UE Motor) and Ability to Manipulate Objects (ARAT)

**Figure 2:**
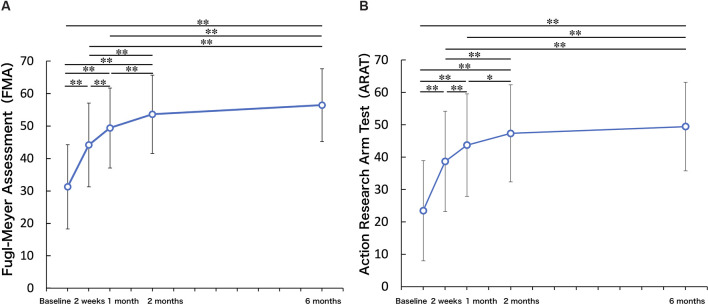
Upper limb function. **(A)** Fugl-Meyer Assessment (FMA). **(B)** Action Research Arm Test (ARAT). Upper limb function and ability to manipulate objects improved up to 2 months and were maintained up to 6 months (One-factor repeated-measures analysis of variance (ANOVA), multiple comparison procedure: Bonferroni correction. ^*^p < 0.05, ^**^p < 0.01).

Figure 2 shows the longitudinal changes in UL function and the ability to manipulate objects. One-way repeated measures ANOVA on FMA-UE motor demonstrated that there was a significant improvement in FMA-UE motor across the 6-month study period (*F* = 91.39, *p* < 0.01). Compared to the FMA-UE motor at T_BL_, FMA-UE motor was significantly increased at T_2w_ (*p* < 0.01), T_1M_ (*p* < 0.01), T_2M_ (*p* < 0.01), and T_6M_ (*p* < 0.01). Compared to the FMA-UE motor at T_2w_, FMA-UE motor was significantly increased at T_1M_ (*p* < 0.01), T_2M_ (*p* < 0.01), and T_6M_ (*p* < 0.01). Compared to the FMA-UE motor at T_1M_, FMA-UE motor was significantly increased at T_2M_ (*p* < 0.01) and T_6M_ (*p* < 0.01), but there was no significant difference between T_2M_ and T_6M_ (*p* = 0.095). Similarly, there was a significant improvement in ARAT across the 6-month study period (repeated-measures ANOVA, *F* = 61.82, *p* < 0.01). Compared to the ARAT at T_BL_, ARAT was significantly increased at the T_2w_ (*p* < 0.01), T_1M_ (*p* < 0.01), T_2M_ (*p* < 0.01), and T_6M_ (*p* < 0.01). Compared to the ARAT at T_2w_, ARAT was significantly increased at T_1M_ (*p* < 0.01), T_2M_ (*p* < 0.01), and T_6M_ (*p* < 0.01). Compared to the ARAT at T_1M_, ARAT was significantly increased at T_2M_ (*p* = 0.01) and T_6M_ (*p* < 0.01), but there was no significant difference between T_2M_ and T_6M_ (*p* = 0.61).

### Sensory Function (FMA-UE Sensory)

The Friedman test on FMA-UE sensory demonstrated that there was a significant improvement in FMA-UE sensory during the 6-month study period (*p* < 0.001). Compared to the FMA-UE sensory at T_BL_, FMA-UE sensory was significantly increased at T_1M_ (*p* = 0.019), T_2M_ (*p* = 0.013), and T_6M_ (*p* < 0.01). Compared to the FMA-UE sensory at T_2w_, FMA-UE sensory was significantly increased at T_6M_ (*p* < 0.05), but there were no significant differences between the other periods.

### Real-World Arm Use

**Figure 3:**
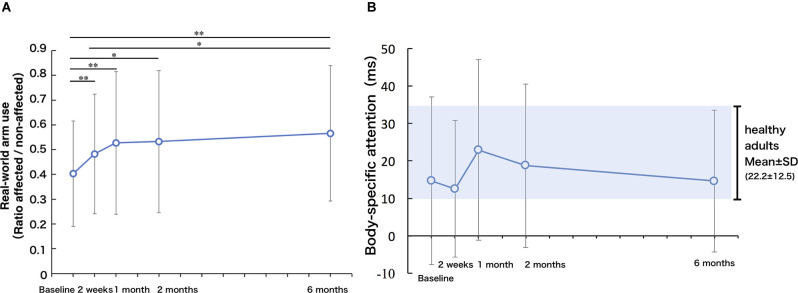
**(A)** Real-world arm use (use ratio). The real-world arm use increased up to 1 month and improved slowly up to 6 months (one-factor repeated-measures ANOVA, multiple comparison procedure: Bonferroni correction. ^*^p < 0.05, ^**^p < 0.01). **(B)** Body-specific attention: longitudinal changes in body-specific attention were highly individual and not significant, but based on mean values, the body-specific attention was maximum at 1 month. The gray area represents the mean ± SD of the index of body facilitation effect in healthy controls.

Figure 3A presents the change in real-world arm use. There was a significant improvement in real-world arm use across the 6 months after baseline (repeated-measures ANOVA, *F* = 9.35, *p* < 0.01). Compared to the real-world arm use at T_BL_, real-world arm use was significantly increased at T_2w_ (*p* < 0.01), T_1M_ (*p* < 0.01), T_2M_ (*p* < 0.05), and T_6M_ (*p* < 0.05). Compared to the real-world arm use at T_2w_, real-world arm use was significantly increased at T_6M_ (*p* < 0.05), while there was no significant difference at T_1M_ (*p* = 0.28) and T_2M_ (*p* = 1.00). There was also no significant difference between T_1M_ and T_6M_.

### Body-Specific Attention to the Paretic Hand

Body-specific attention to the paretic limb did not change significantly over the 6 months after baseline (repeated-measures ANOVA, *F* = 1.30, *p* = 0.28, Figure 3B). Compared with the previously published data on a healthy adult group (Aizu et al., 2018), stroke participants showed significantly lower body-specific attention to the paretic hand at T_2w_ (*p* < 0.05, *t* value = 2.20, 95% CI 0.9–20.1). There was a high degree of variability in body-specific attention across participants. The mean value of body-specific attention to the paretic limb peaked at T_1M_ (approaching the mean value of healthy adults), and then declined slightly between T_1M_ and T_6M_, but remained within the range of the values of healthy adults at T_6M_ (no significant difference compared to healthy older adults).

The average incorrect response was 0.45 times in the catch trials (16 trials), and the average response of exclusions due to the patient’s exceeding the 2SD was 1.42 times in all trials (80 trials).

### Correlation Between Body-Specific Attention and Real-World Arm Use

#### Correlation Analysis of Longitudinal Data

**Figure 4:**
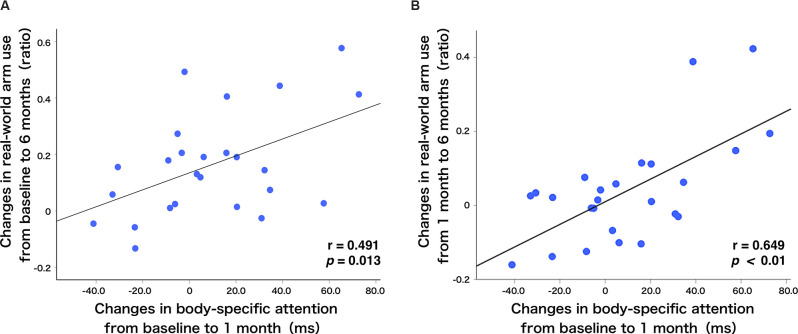
Relationship between real-world arm use and body-specific attention. **(A)** There was a positive correlation between the changes in body-specific attention from baseline to 1 month and the changes in the real-world arm use from baseline to 6 months (*r* = 0.491, *p* = 0.013). **(B)** Additionally, there was a positive correlation between the changes in body-specific attention from baseline to 1 month and the changes in the real-world arm use from 1 month to 6 months (*r* = 0.649, *p* < 0.01). The blue dots represent individual patients.

**Figure 5:**
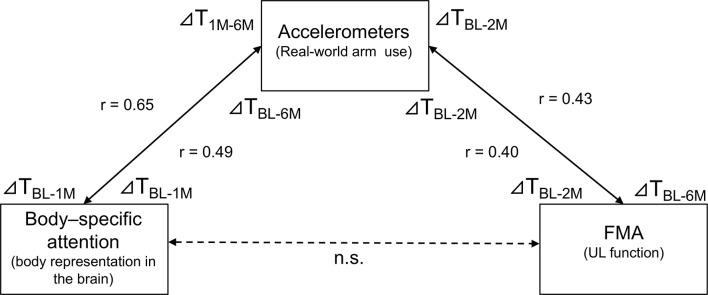
Relationship between changes in body-specific attention, real-world arm use, and UL function as revealed by correlation analysis using longitudinal data. There was a significant positive correlation between the amount of change in body-specific attention from baseline to 1 month and the change in real-world arm usage from baseline to 6 months (*r* = 0.491, *p* = 0.013). Moreover, there was a significant positive correlation between the amount of change in body-specific attention from baseline to 1 month and the change in real-world arm use from 1 month to 6 months (*r* = 0.649, *p* < 0.01). The amount of change in real-world arm use from baseline to 2 months had a significant positive correlation with the amount of change in UL function over the same period (*r* = 0.40, *p* = 0.048). In addition, the amount of change in real-world arm use from baseline to 2 months had a significant positive correlation with the amount of change in UL function from baseline to 6 months (*r* = 0.43, *p* = 0.034). On the other hand, there was no correlation between body-specific attention and UL function. T: Time of measurement, BL: baseline, M: month, (ex. ΔT_BL_-_6M_: during the measurement period from baseline to 6 months).

To show alterations in the relationship between the amount of change in body-specific attention and the amount of change in real-world arm use over time visually, Figure 4 presents individual plots from the study participants. There was a significant, moderate, positive relationship between the amount of change in body-specific attention from T_BL_ to T_1M_, and in the amount of change in real-world arm use from the T_BL_ to T_6M_ (*r* = 0.491, *p* = 0.013, Figures 4A, 5). This relationship increased between the amount of change in body-specific attention from T_BL_ to T_1M_ and the amount of change in the real-world arm use from T_1M_ to T_6M_ (*r* = 0.649, *p* < 0.01, Figures 4B, 5), indicating that long-term recovery of real-world arm use was associated with body-specific attention to the paretic limb in the early phase after stroke. Moreover, there was a significant, moderately positive relationship between the amount of change in body-specific attention from T_BL_ to T_2w_ (the period of least longitudinal change in body-specific attention) and the amount of change in real-world arm use from T_BL_ to T_2M_ (*r* = 0.42, *p* = 0.035).

In addition, correlations between the amount of change in body-specific attention and individual factors and clinical outcomes were confirmed. There were no correlations with age at enrollment, duration after stroke onset, UL function (FMA-UE motor), sensory impairment (FMA-UE sensory), and ability to manipulate objects (ARAT).

Regarding the association between the amount of change in real-world arm use and other individual factors, in the short-term, there was a significant positive correlation between the amount of change in real-world arm use from T_BL_ to T_2M_ and the amount of change in FMA-UE motor from T_BL_ to T_2M_ (*r* = 0.40, *p* = 0.048, Figure 5). Furthermore, in the long-term, there was a moderately significant positive correlation between the amount of change in real-world arm use from T_BL_ to T_2M_ and the amount of change in FMA-UE motor from T_BL_ to T_6M_ (*r* = 0.43, *p* = 0.034, Figure 5). The amount of change in real-world arm use during the early phase showed results associated with short- and long-term changes in UL function. No correlations were found with age at enrollment, duration since onset, sensory impairment (FMA-UE sensory), and ability to manipulate objects (ARAT).

#### Correlation Analysis of Cross-sectional Data

In the cross-sectional correlation analyses, there were no correlations between body-specific attention and other factors at each time point from T_BL_ to T_6M_. Although there was a strong positive correlation between real-world arm use and FMA-UE motor at each time point from T_BL_ to T_6M_, the relationship gradually weakened with time after onset (T_BL_ *r* = 0.78, *p* < 0.001; T_2w_ *r* = 0. 68, *p* < 0.001; T_1M_ *r* = 0.65, *p* < 0.001; T_2M_ *r* = 0.59, *p* = 0.002; T_6M_ *r* = 0.59, *p* = 0.002). In addition, there was positive correlation between real-world arm use and ARAT at each time point from T_BL_ to T_6M_ (T_BL_ *r* = 0.59, *p* = 0.002; T_2w_ *r* = 0. 65, *p* = 0.001; T_1M_ *r* = 0.61, *p* = 0.001; T_2M_ *r* = 0.54, *p* = 0.005; T_6M_ *r* = 0.55, *p* = 0.005).

## Discussion

In this study, we elucidated the longitudinal changes in body-specific attention to the paretic limb and real-world arm use in 25 stroke patients with hemiparesis, using psychophysical methods and accelerometers, respectively. The relationship between body-specific attention to the paretic limb and real-world arm use was also examined. To the best of our knowledge, no previous study had examined the longitudinal changes in body-specific attention to the paretic limb and real-world arm use in stroke patients, or the relationship between them. Our results showed that UL function and real-world arm use improved significantly across the 6-month study period. The longitudinal change in body-specific attention to the paretic hand was greatest at 1 month, although there were large individual differences. Our study also showed that, in patients in whom body-specific attention to the paretic hand increased up to 1 month, the real-world arm use increased up to 6 months. In addition, an increase in real-world arm use was associated with the recovery of UL function. Thus, our data suggested that increased body-specific attention to the paretic hand in the early phase after stroke was associated with long-term recovery of real-world arm use. These findings provide new insights into the relationship between real-world arm use and body representation in the brain, which may facilitate the use of the paretic hand, leading to long-term UL recovery after stroke.

### Longitudinal Changes of Real-World Arm Use

One of the new findings in this study was that real-world arm use increased in the early phase after stroke and continued to improve over 6 months. This result was in contrast to previous studies that showed no improvement in daily use of the paretic arm, despite improved UL function, in subacute (Rand and Eng, 2012) and chronic stroke patients (Rand and Eng, 2015; Waddell et al., 2017), and supports recent studies that have shown improvement in arm use in the early phase (first 3 months) after onset (Waddell et al., 2019a). Additionally, our results confirmed the long-term recovery process up to 6 months. The present study also showed that UL function and the ability to manipulate objects during the process of recovery from UL paralysis after stroke improved significantly up to 2 months, and the improvement was maintained up to 6 months. These longitudinal data support previous reports on the motor recovery process in post-stroke UL paralysis (Duncan et al., 2000).

Participants had a mean FMA-UE motor of 31.3 at enrollment, which is a relatively severe score that would classify them as moderately paralyzed according to the FMA-UE motor severity classification (Severe: 0–28; Moderate: 29–42; Mild: 43–66; Woytowicz et al., 2017). The real-world arm use ratio at 6 months was 56.5%, which is a high score considering the previously reported 35% use ratio at 12 months after stroke (Rand and Eng, 2015). In most previous observational studies using accelerometers, the content and amount of UL rehabilitation after stroke were not controlled. Such differences in the rehabilitation provided may have affected the amount of use of the paretic hand. In the present study, by using a standardized program for the content and amount of UL rehabilitation after stroke (Harris et al., 2009, 2010; Murdolo et al., 2017; Simpson et al., 2017), the variation in arm use due to different rehabilitation methods was minimized and certain enhancing effects on arm use were achieved as compared to previous studies. Previous clinical trials of this program have reported improvements in UL function (Chedoke Arm and Hand Activity Inventory, ARAT, grip strength) and use of the arm in daily life (Harris et al., 2009). However, those previous studies used the Motor Activity Log (MAL), a patient-reported assessment for arm use, and it was unclear whether real-world arm use measured objectively also improved (Harris et al., 2009). In the present study, we demonstrated for the first time the process of long-term recovery of real-world arm use over 6 months, as observed with accelerometers, using a program that standardizes the content and amount of UL rehabilitation after stroke.

### Longitudinal Changes of Body-Specific Attention

Body-specific attention showed a different recovery process from that of recovery of UL function and real-world arm use. Longitudinal changes in body-specific attention were highly individual and not significant, but based on mean values, they increased during the early post-stroke phase, up to 1 month, and decreased slowly up to 6 months. When compared with previous data of the body-specific attention for healthy older subjects (Aizu et al., 2018), body-specific attention in the present study was significantly lower at 2 weeks and did not differ from the values of healthy subjects at 1 month. Since body-specific attention is calculated based on the body facilitation effect of visual detection on the self-hand (Reed et al., 2006, 2010; Tseng et al., 2012), the fact that it was significantly lower than that of the healthy elderly subjects at the 2 weeks means that the paretic hand was not properly represented in the brain. In contrast, the fact that body-specific attention was not significantly different from that of the healthy elderly subjects at 1 month means that the paretic hand was properly represented in the brain as in the healthy elderly subjects. Previous fMRI studies examining brain representations of hand-related visual targets have reported greater activation in the intraparietal sulcus (IPS) and lateral occipital complex (LOC) when the visual target was presented near the hand compared to when it was presented farther away (Makin et al., 2007). Furthermore, comparisons between the real and dummy hand and different hand positions revealed that activity in the posterior IPS and LOC was more modulated by visual information of the hand, while activity in the anterior IPS was more modulated by proprioceptive information of the hand (Makin et al., 2007). The authors reported that cortical areas in the posterior IPS and LOC may represent the hand-centered space mainly visually, while the anterior IPS may represent the space around the hand multisensory. It has been reported that such peripersonal spatial representation is represented by the posterior parietal cortex (PPC), which includes the IPS (Moseley et al., 2012). The PPC has a role in multisensory integration of vision and somatosensory perception and is classically considered to be a region involved in body schema and body image, which is appropriate for the representation of the self-body in the brain. The PPC is also involved in sensorimotor transformations and in directing attention to important information for motor planning to reach an object of interest based on body representations (Corbetta and Shulman, 2002; Sestieri et al., 2017). The results of these studies also suggest that measuring body-specific attention using a visual stimulus detection task may explain changes in body representations in the brain. Therefore, it is suggested that the increase in body-specific attention observed in the early phase reflects a process of adaptive changes in the body representation of the paretic hand in the brain.

Although body-specific attention at 6 months in stroke patients was not significantly different from that of healthy older people, the mean score was lower than that at 1 month after enrollment. Our previous cross-sectional study (Aizu et al., 2018) showed a negative correlation between body-specific attention and interval from stroke onset (7–192 months), suggesting that body-specific attention may gradually decrease during the stage that patients learn not to use the paretic hand, a phenomenon known as learned nonuse (Taub et al., 2002). The present longitudinal study supports such maladaptive changes of body-specific attention in chronic patients with stroke, suggested by the cross-sectional study.

Most patients have to adapt the body representations in the brain that have been constructed over a long period of time to a body that has changed significantly and suddenly due to stroke. While mildly paralyzed patients may adapt early, many paralyzed patients experience varying degrees of mismatch between their body and body representations in response to sudden changes in the body and nervous system. Similarly, a mismatch is known to occur in amputees with phantom limb pain, who do not have direct brain damage, but where the mismatch is caused by an inability to update the body representations previously built up in the brain in response to sudden physical changes (Flor et al., 1995; Ramachandran and Hirstein, 1998). Such mismatch between body representation and real body is also likely to occur in stroke patients, which might lead to maladaptive changes of body representation or a state known as learned nonuse. In addition, it has been reported that in stroke patients, early after the onset of stroke, the decrease in intracortical inhibition, or disinhibition (Liepert et al., 2000; Shimizu et al., 2002; Manganotti et al., 2008) and adaptive or compensatory reorganization of dynamic brain functions occur in the bilateral cerebral hemispheres (Marshall et al., 2000; Feydy et al., 2002; Ward et al., 2003; Tombari et al., 2004; Rehme et al., 2011b), which is considered to be one of the factors explaining the process of changes in body representation in the brain. Some animal studies (Calford and Tweedale, 1988, 2009) and neuroimaging studies in patients (Simões et al., 2012) suggested that plastic changes in brain sensory maps and body representations can occur either immediately (Calford and Tweedale, 2009) or slowly (Grüsser et al., 2001; Simões et al., 2012). Thus, longitudinal changes in body-specific attention were not significant, but based on mean values, the fast and large changes observed in the early phase up to 1 month and the slow and gradual changes observed up to 6 months in the present study may reflect the process of adaptive or maladaptive changes in the body representation of the paretic hand in the brain.

Body representations in the brain are formed and updated by sensory information from the body, and have a significant impact on motor control and body perception (Aymerich-Franch and Ganesh, 2016; Naito et al., 2016; Oouchida et al., 2016; Matamala-Gomez et al., 2020). Based on this principle, body-specific attention, which reflects body representations in the brain, is updated by sensory input during arm use, so it is conceivable that the more upper limbs are used, the higher the body-specific attention. Contrary to this relationship, it is important to clarify whether an increase in body-specific attention contributes to the promotion of arm use in order to develop rehabilitation that promotes the arm use. Symptoms characterized by reduced or complete lack of awareness and response to body or spatial stimuli contralateral to the brain lesion that occur after a stroke are known as USN (Buxbaum et al., 2004). In particular, neglect symptoms related to the body are referred to as body representation neglect, and deficits in attention to the body are recognized as body representation problems (Glocker et al., 2006; Cocchini et al., 2010). A previous report examining the relationship between attention to the paretic side and UL function after stroke showed that the more severe the USN, the lower the improvement in UL function (FMA), and reported on the possibility that the lack of attention to the paretic side space contributed to the nonuse of the paretic arm (Nijboer et al., 2014). Conversely, limb activation training (Robertson et al., 2010), which involves active paretic arm use in practice and daily life, has been reported to improve body-related deficits in spatial neglect in subacute stroke patients (Reinhart et al., 2012). It was concluded that the improvement in performance was probably due to activation of body representation in the brain by limb activation training (Reinhart et al., 2012). Furthermore, patients with motor neglect, a symptom of refusal to move the paretic arm, have both motor intention and attentional problems. However, it has been reported that when attention is directed to the paretic hand, motor performance improves (spatial errors during movement are improved; Punt et al., 2013). Based on these findings, it was known that attention to the paretic side space was interrelated with UL motor performance and arm use, and that increased attention to the paretic side space affected the paretic arm use. However, it is not clear whether increased body-specific attention would increase paretic arm use in stroke patients. The present study demonstrated positive correlation between increase in body-specific attention and increase in paretic arm use for the first time.

### Correlation Between Body-Specific Attention and Real-World Arm Use

The most salient finding of this study was that there was a positive correlation between the amount of change in body-specific attention during the early phase (longitudinal changes in body-specific attention were most remarkable up to 1 month) and the amount of long-term change in real-world arm use. These results were consistent with our hypothesis. Specifically, there was a positive correlation between the amount of change in body-specific attention (from baseline to 1 month) and the amount of change in real-world arm use (from baseline to 6 months) in this study. Furthermore, there was a stronger positive correlation between the amount of change in body-specific attention over the same period and the amount of change in real-world arm use (from 1 month to 6 months). Although short-term, there was also a positive correlation between the amount of change in body-specific attention at 2 weeks from baseline and the amount of change in real-world arm use at 2 months from baseline, supporting the idea that adaptive changes in body-specific attention are associated with increased real-world arm use. In contrast, cross-sectional correlation analysis showed no correlation between body-specific attention and real-world arm use in the subacute (baseline, 2 weeks, 1 month, 2 months) to early chronic post-stroke (6 months) stages in this study. These findings suggest that body-specific attention to the paretic hand would be an enhancing factor for arm use in hemiparetic patients.

Our results showed that changes in body-specific attention up to 1 month were associated with long-term changes in real-world arm use but changes in body-specific attention after 2 months were not associated with real-world arm use. In the early post-stroke period, previous fMRI and TMS studies have shown brain plastic changes, including activation of the contralesional hemisphere (Marshall et al., 2000; Feydy et al., 2002; Ward et al., 2003; Tombari et al., 2004; Rehme et al., 2011b), disinhibition of the ipsilesional hemisphere (Liepert et al., 2000; Manganotti et al., 2008), and disinhibition of the contralesional hemisphere (Shimizu et al., 2002; Manganotti et al., 2008). Furthermore, brain reorganization through dynamic changes in functional connectivity has been reported in both task and resting state fMRI studies (Grefkes et al., 2010; Grefkes and Fink, 2011; Park et al., 2011; Rehme et al., 2011a). In addition, long-term changes in brain activity reported in longitudinal studies have also revealed a process of convergence of bilateral hemispheric activity observed in the early post-onset period to the ipsilesional hemisphere between the acute and chronic periods (Marshall et al., 2000; Feydy et al., 2002; Ward et al., 2003; Tombari et al., 2004), as well as a process of asymmetry in intercortical functional connectivity that was maximal at 1 month but slowly reduced by 6 months (Park et al., 2011). These processes of brain disinhibition and long-term changes in brain activity and functional connectivity after stroke in previous studies are generally consistent with the process observed in the present study, in which body-specific attention, which reflects body representations in the brain, is maximal at 1 month and slowly decreases by 6 months.

Although the neural basis of body-specific attention is still unclear, there is evidence that body representation neglect (Glocker et al., 2006; Cocchini et al., 2010) and pusher phenomenon, which are impairments of body representations in the brain, often improve early after onset (Karnath et al., 2002; Danells et al., 2004). Such reports also support the possibility that reconstruction of body representations in the brain may occur early after stroke. In addition, it has been shown that reorganization of brain networks in the early post-stroke period promotes recovery of motor functions after stroke (Volz et al., 2016), which emphasizes the importance of brain reorganization in the early post-stroke period. Furthermore, it has been shown that the recovery of brain networks also correlates with the recovery of higher-order functions such as attention and language (Siegel et al., 2018), and it is possible that these brain networks are involved in the recovery of body-related attention. In the present study, we found an association between increased body-specific attention and improved long-term real-world arm use during a period of brain reorganization involving cortical disinhibition and dynamic changes in intracortical and intercortical functional connectivity. Therefore, it is suggested that the increase in body-specific attention (adaptive changes in body representations in the brain) during the period of abundant plasticity in the brain may have facilitated arm use based on appropriate body representations.

As expected from the principle of use-dependent brain plasticity reported in previous studies (Taub et al., 2002; Gauthier et al., 2008), early (from baseline to 2 months) changes in real-world arm use were correlated positively with changes in FMA-UE motor during the same period and also the longer period (from baseline to 6 months). Thus, it is concluded that real-world arm use is associated with long-term improvement in UL function.

In the present study, we showed that increased body-specific attention to the paretic hand in stroke patients, i.e., adaptive changes in body representation of the paretic hand in the brain, is associated with increased real-world arm use. Some studies have shown that improvement of paretic hand use contributes more to the improvement of quality of life (QOL) in stroke patients than UL function (Kelly et al., 2018). This underscores that understanding the relationship between body representations in the brain and real-world arm use has clinical significance for improving QOL in stroke patients.

### Limitations

There are several limitations to this study. First, this study used GRASP to control the content and amount of upper extremity rehabilitation, and the results were obtained under the condition of 1 h of practice per day during hospitalization. Therefore, the results may not be the same for patients with poor UL practice. However, previous observational studies that focused on real-world arm use could not exclude the influence of differences in the content and amount of rehabilitation on the amount of real-world arm use. Thus, GRASP was suitable for this study because it could provide a standardized rehabilitation program according to the severity of paralysis, and a wide range of patients with severe to mild paralysis could be targeted. Second, only univariate analysis was available to analyze the association among arm use, body-specific attention, and UL function, as multivariate analysis was judged inappropriate due to the small sample size. Third, this was a single-center study. It is necessary to conduct a multicenter study in the future, with more case accumulation. In addition, the above results were obtained only up to 6 months, and a study with a longer period of observation would be warranted because plastic changes in the brain have been reported even after 6 months (Tombari et al., 2004; Lin et al., 2018). Finally, further studies are also needed to discover ways to increase body-specific attention to the paretic limb in order to examine the effects of the increase in body-specific attention on real-world arm use and motor recovery after stroke.

## Conclusion

In the recovery process of paretic UL, body-specific attention to the paretic hand was found to be related to real-world arm use in patients after stroke. The findings in this longitudinal study suggest adaptive changes in body representation of the paretic limb in the brain during the early phase contributes to increasing long-term real-world arm use and are associated with UL recovery through use-dependent plasticity. We believe that the results will contribute to the innovation of rehabilitation strategies to enhance adaptive changes in body representation in the brain and increase real-world arm use after stroke.

## Data Availability Statement

The raw data supporting the conclusions of this article will be made available by the authors, without undue reservation.

## Ethics Statement

The studies involving human participants were reviewed and approved by The Tohoku University and Yamagata Saisei Hospital ethics committee. The patients/participants provided their written informed consent to participate in this study.

## Author Contributions

ST and YS recruited patients and were medically in charge of the patients. RO managed the quality of clinical rehabilitation and examinations; collected the clinical examination data and analyzed the clinical examination data. RO, YO, NA, TS, HS, and S-II conceived and designed the study. YO and S-II critically reviewed the manuscript. All authors contributed to the article and approved the submitted version.

## Conflict of Interest

The authors declare that the research was conducted in the absence of any commercial or financial relationships that could be construed as a potential conflict of interest.

## Publisher’s Note

All claims expressed in this article are solely those of the authors and do not necessarily represent those of their affiliated organizations, or those of the publisher, the editors and the reviewers. Any product that may be evaluated in this article, or claim that may be made by its manufacturer, is not guaranteed or endorsed by the publisher.
